# Establishment and Verification of Multiaxis Fatigue Life Prediction Model

**DOI:** 10.1155/2021/8875958

**Published:** 2021-02-02

**Authors:** Zhuo Fu, Xiang Li, Sha Zhang, Hanqing Xiong, Chi Liu, Kun Li

**Affiliations:** ^1^Department of Mechanical and Electronic Engineering, Changsha University, Changsha 410022, China; ^2^Department of Mechanical and Electronic Engineering, Central South University, Changsha 410083, China

## Abstract

A fatigue life prediction model with multiaxis load is proposed. The model introduces a new effective cyclic parameter, equivalent stress on the critical surface, to modify the Suntech model. The new damage parameters are not related to empirical constants, hence more applicable for practical application in engineering. The multiaxis fatigue test was carried out with high-strength aluminum alloy 7075-T651, and the multiaxis fatigue life prediction of the test piece was conducted with the finite element software. The experiment result shows that the model proposed is effective for predicting the fatigue life under multiaxis load.

## 1. Establishment of Multiaxis Fatigue Life Prediction Model

Most mechanical equipment and engineering structural parts run in a complex multidimensional stress state practically. Even if they are under a uniaxial load, the local area is still in the multiaxis stress state due to their complex geometric shape [1]. The interaction of multiaxial stress is the main reason for the fatigue failure of mechanical equipment and structural parts. Therefore, it is of great value to study the multiaxis fatigue life prediction method. To date, a variety of multiaxis fatigue life prediction models [2–6] has been proposed. However, suffering from the limited experiment conditions, the complexity of loading form, and the diversity of materials [7], all the above works are not able to predict multiaxis fatigue life under different loading conditions accurately [8].

The current multiaxial fatigue life prediction mostly adopts the critical plane method, which uses the maximum strain shear plane as the damage plane of the material. The maximum shear strain *γ*_max_ and the normal strain *ε*_*n*_ on the plane are considered to contribute to the fatigue damage of the material. The critical plane method is used to predict the multiaxis fatigue life. When there is a phase difference between the tensile strain and the shear strain of a material or object, that is, when it is subjected to nonproportional loading, the strain principal axis of the material or object will periodically change with the cyclical change of the loading. The cyclical change of the strain principal axis results in that the shear strain and the normal strain on the critical plane cannot reach the maximum simultaneously, which leads to the additional strengthening effect of the material. Studies show that the bigger the phase difference angle between the tensile and torsional stresses is, the more prominent the additional effect of the strengthening is while the lower the fatigue life of the material is. Reference [9] proposed a new parameter, the normal strain, which considers that the magnitude of the normal strain amplitude between the two maximum shear strain return points is an important factor affecting the fatigue crack propagation, and meanwhile, the normal strain range can better describe the nonproportional cycle additional hardening effect of the material under the fatigue life of the phenomenon and provided the following multiaxis fatigue damage parameter model as follows:

(1) $$\begin{array}{c} \frac{\varepsilon_{\text{eq}}}{2}=\sqrt{\left(\varepsilon_{n}^{*2}\right)+\frac{1}{3}{\left(\frac{{\Delta \gamma }_{\max}}{2}\right)}^{2}}=\frac{\sigma_{f}^{\prime }}{E}{\left(2N_{f}\right)}^{b}+\varepsilon_{f}^{\prime }{\left(2N_{f}\right)}^{c}, \end{array}$$

where the normal strain equation

(2) $$\begin{array}{c} \varepsilon_{n}^{*}=0.5\times \varepsilon_{n}\left(1+\cos\varphi \right). \end{array}$$

**Figure 1:**
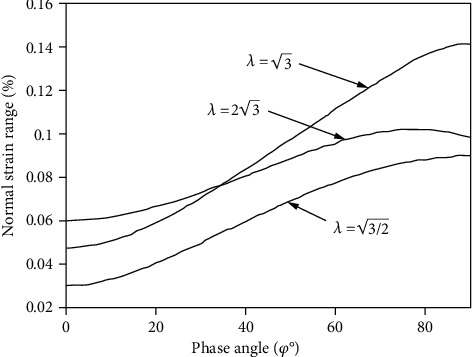
Relationship between normal strain range and phase angle.

When the equivalent strains have the same values but the ratio of shear strain to normal strains is different, the normal strain of the material varies with the phase angle, as shown in Figure 1. Figure 1 reveals the roughly positive correlation between the phase difference angle and the normal strain of the material, and the strain change is obviously larger. More detailedly, the normal strain of the material is not monotonically increasing in the range, but with the increase of the phase difference angle, it increases first and then decreases, and therefore, normal strain is not very good to describe the additional stiffening effect of the material at low strain rates.

**Figure 2:**
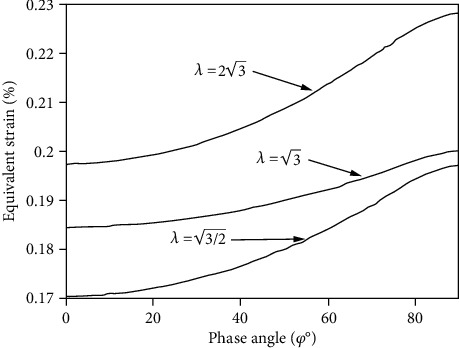
The relationship between the equivalent strain and phase angle of the model.

Figure 2 depicts the relationship between the equivalent strain calculated from the Suntech model and the phase difference angle under the condition that the standard deviation of shear strain and positive strain is 0.4% and the ratio of shear strain to normal strain is different. When the phase difference angle increases, the Suntech model damage parameter also increases.

However, according to Figure 1, the normal strain of the damage parameter does not increase with the rise of the phase difference angle at the low strain ratio. Therefore, the model cannot describe the multiaxis fatigue life well under the low strain ratio. From the microscopic point of view, the fatigue crack usually occurs in the local plastic zone of the slip zone. The fatigue crack growth is the polymerization process along the crack tip shear zone. The normal strain and the normal stress on the crack surface make this polymerization accelerate, contributing to the growth of fatigue crack, so it should be considered that the normal strain and normal stress have effect on fatigue damage accumulation. When the equivalent strain of the material is the same size while the loading method is different, the fatigue life of the material will have some differences. Correspondingly, as a multiaxis fatigue damage parameter, only the equivalent strain combined with the shear strain and the normal strain on the critical plane has some limitations.

Therefore, in the multiaxis fatigue loading process, besides the contribution of strain on fatigue damage, the critical surface of the stress, to a certain extent, accelerates the formation and expansion of fatigue cracks. So, the multiaxis fatigue life prediction should consider the impact of not only strain on fatigue damage but also critical surface stress. The stress on the critical plane can be obtained by the Osgood-Ramberg equation:

(3) $$\begin{array}{c} \frac{\Delta \varepsilon_{\text{eq}}}{2}=\frac{\Delta \sigma_{\text{eq}}}{2E}+{\left(\frac{\Delta \varepsilon_{\text{eq}}}{2K^{\prime }}\right)}^{1/n^{\prime }}, \end{array}$$

where *K*′ is the cyclic strength factor and *n*′ is the cyclic strain hardening factor.

**Figure 3:**
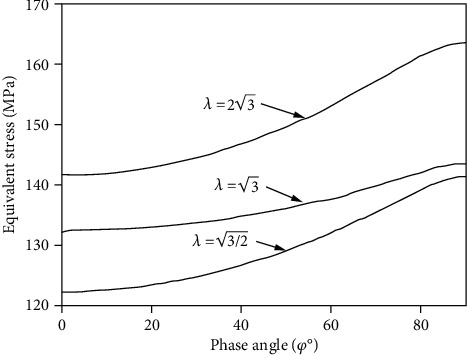
The relationship between equivalent stress and phase angle in critical plane.

Figure 3 depicts the relationship between the equivalent stress on the critical plane and the phase difference angle under the condition that the standard deviation of shear strain and positive strain is 0.4% and the ratio of shear strain to normal strain is different. The equivalent stress and the retard angle on the critical plane show a monotonically increasing relationship under the condition that the strain ratio is different and reach the maximum value in 900 nonproportional loading, which is in accordance with the actual fatigue life, with the fact that the phase difference angle decreases. Comparing with Figure 2, the equivalent stress on the critical plane is used to describe the effect of the nonproportional additional effect on the multiaxial fatigue life.

In this paper, based on the existing multiaxis fatigue life prediction model, considering the different loading conditions, the equivalent stress on the critical plane is used to describe the nonproportional additional effect, and a new multiaxis fatigue life prediction model

(4) $$\begin{array}{c} \Delta \sigma_{\text{eq}}\frac{\Delta \varepsilon_{\text{eq}}}{2}=\left(1+\upsilon_{e}\right)\frac{{\left(\sigma_{f}^{\prime }\right)}^{2}}{E}{\left(2N_{f}\right)}^{2b}+\left(1+\upsilon_{p}\right)\sigma_{f}^{\prime }\varepsilon_{f}^{\prime }{\left(2N_{f}\right)}^{b+c}, \end{array}$$

where *N*_*f*_ is fatigue life, *σ*_*f*_′ is fatigue strength coefficient, *ε*_*f*_′ is fatigue ductility coefficient, *b* is fatigue strength index, and *c* is the fatigue ductility index.

The material parameters of Equation (4) can be obtained by the uniaxial fatigue test. It is also possible to estimate the fatigue material constants by means of the relevant manual and literature, which can be used to estimate the multiaxis fatigue life.

## 2. Multiaxis Fatigue Test

### 2.1. Experimental Materials

High-strength aluminum alloy 7075-T651 was employed as the study object, the chemical composition and fatigue properties of which are shown in Tables 1 and 2.

### 2.2. Experimental Equipment

**Figure 4:**
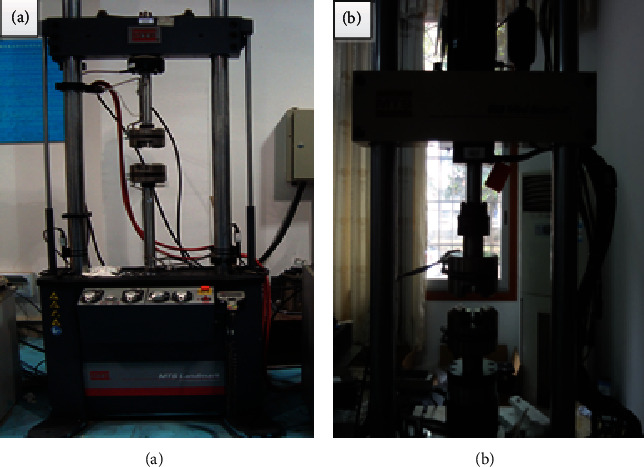
Electrohydraulic servo fatigue testing machine.

Fatigue tests were carried out on two different fatigue testing machines, depending on the loading method. The uniaxial tensile fatigue test was carried out on an MTS-50Kg fatigue tester. In the MTS-858 is the pull-twist composite load fatigue test.  Figure 4(a) is MTS-50KN, and Figure 4(b) is the MTS-858 electrohydraulic servo fatigue test system.

### 2.3. Specimen Shape

**Figure 5:**
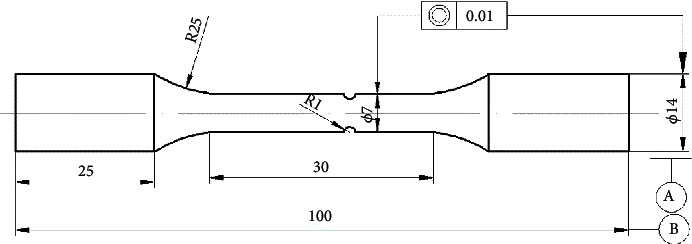
Schematic diagram showing the shape and size of fatigue specimen.

The test piece is made of bar nuts, and the notch is a circular groove with a radius of 1 mm at the center of the specimen. The specific shape and dimensions of the specimen are shown in Figure 5. In order to eliminate cutting marks generated in the machining process and the aluminum surface's protective film, the surface of the bar was polished using fine sandpaper before the test.

### 2.4. Experimental Results

The loading of the material during the test and the detailed experimental results are shown in Table 3.

**Figure 6:**
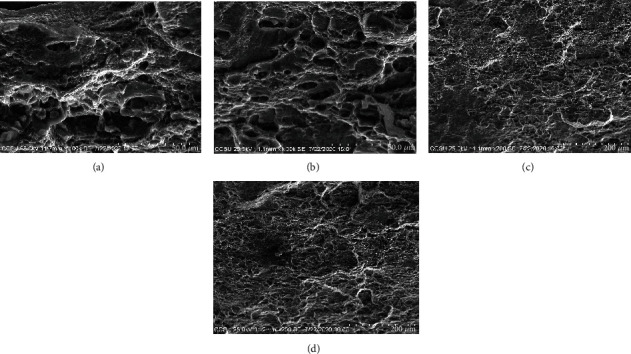
Scanning electron microscopy.

Detected by the Hitachi SU1500 scanning electron microscope [10, 11], the resolution of the scanning electron microscope is 50 *μ*m and 200 *μ*m, respectively, and the results are shown in Figures 6(a)–6(d).

## 3. Finite Element Simulation

**Figure 7:**
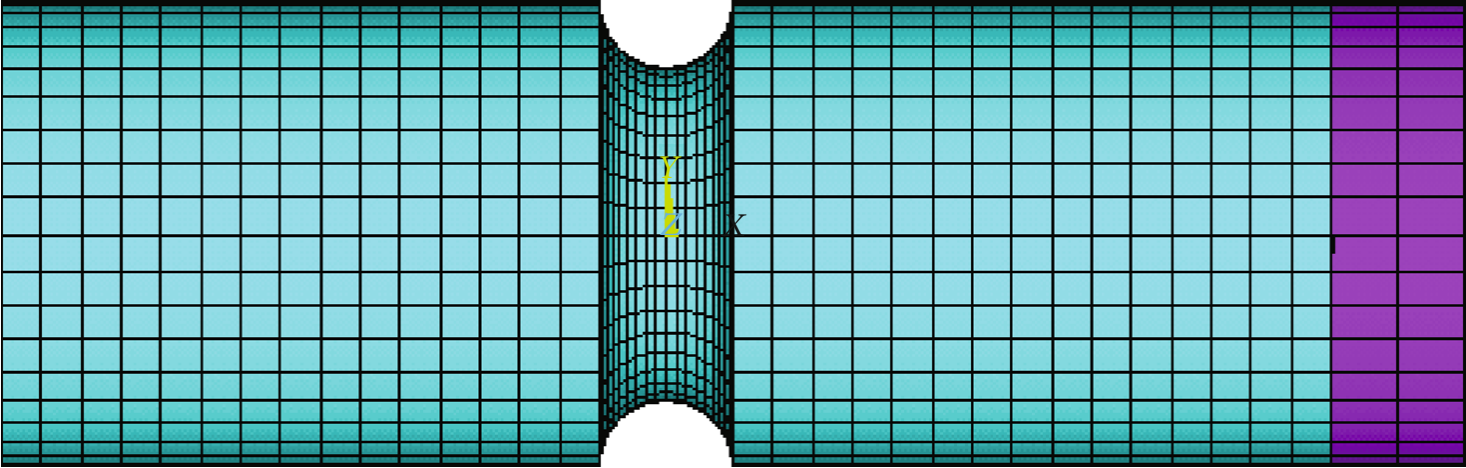
Finite element grid.

It is overcomplicated to calculate the stress and strain state of the notched using the theoretical formula. In order to obtain the stress and strain state of the material quickly and effectively, the finite element analysis software is used to solve the problem. In order to make the simulation closer to the test conditions, the effective distance of the finite element model is set to 20 mm to match the effective length of the extensometer during the test. At the same time, for the convenience of loading, a 2 mm long rigid body is established at the right end of the model, and the tensile and torsional state of the finite element model is realized by loading on the rigid body. When meshing, the two-dimensional grid is firstly meshed and then rotated to generate a three-dimensional grid. The grid is divided as shown in Figure 7, where there are 30565 nodes in the range. The number of units is 15504, by which the purple part of the elastic modulus takes 108 MPa, its default for the rigid body.

The finite element analysis method is used to simulate the loading condition under the experimental conditions, and the stress and strain states at the point of danger and the corresponding damage parameter are obtained as shown in Table 4.

As Table 4 shows, in the case of proportional and unidirectional loading, the risk point of the specimen is the same node. Under nonproportional loading conditions, the risk of the specimen is transferred to the other nodes in the notch position.

**Figure 8:**
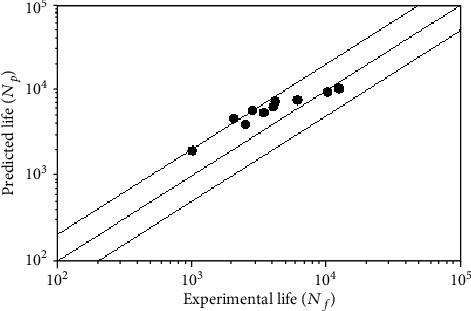
Life prediction results for missing parts.

The results show that the damage parameters recorded in Table 4 are brought into the multiaxial fatigue life prediction model, and the multiaxial fatigue life of the missing parts under different loading conditions is obtained. The results of the prediction are shown in Figure 8.

Figure 8 describes the relationship between the predicted life of the notch and the experimental life, where the most solid line represents the case where the test result is consistent with the predicted result. The two solid lines on the edge indicate that the predicted result is different from the experimental result, times the error factor. As can be seen from the figure, the prediction results are 70% (7) located within twice the error factor; the prediction accuracy is higher. At the same time, the predicted life is basically higher than the experimental life. The reason may be that the actual stress in the sample gap is much more concentrated than in the ideal model.

## 4. Results

1. The results show that the normal strain on the critical plane cannot reflect the effect of the nonproportional cycle additional strengthening effect on the fatigue life under the low strain ratio
2. The new model of life prediction and experimental results of the comparison show that the new multiaxis life prediction model has a better prediction accuracy. The multiaxis fatigue life prediction model established in this paper is free of empirical constants and is easy to be used in engineering

## Figures and Tables

**Table 1:** Chemical composition of 7075 aluminum alloys (mass fraction %).

Zn	Mg	Cu	Cr	Fe	Si	Mn	Ni	Al
5.1-6.1	2.1-2.9	1.2-2.0	0.180-28	≤0.50	≤0.40	≤0.30	≤0.05	Margin

**Table 2:** Uniaxial tensile fatigue properties of materials.

*σ*_*f*_′ (MPa)	*b*	*ε*_*f*_′	*c*	*K*′	*n*′
1576	-0.1609	0.1575	-0.6842	747	0.0597

**Table 3:** Fatigue results of notch pieces.

Specimen number	Loading conditions		Phase angle (*φ*^0^)	Life *N*_*f*_
	Stretch (kN)	Twist (N∗m)		
1-1	5.5	0	0	12455
1-2	5.6	0	0	10240
1-3	5.8	0	0	6152
1-4	6.0	0	0	4056
1-5	6.2	0	0	3485
1-6	6.5	0	0	2545
2-1	3.5	10	0	2854
2-2	3.0	9	0	4218
2-3	3.5	10	90	1524
2-4	3.0	9	90	2058

**Table 4:** Stress and strain states of the specimen at the point of danger.

Number	Dangerous node number	Stress (MPa)	Strain (%)	Shear strain (%)	Damage amount
1-1	175	336.577	0.5612	0.1554	1.913
1-2	175	342.679	0.5714	0.1582	1.983
1-3	175	354.936	0.5918	0.1639	2.127
1-4	175	367.175	0.6122	0.1695	2.2764
1-5	175	379.414	0.6326	0.1752	2.40
1-6	175	397.773	0.6632	0.1836	2.674
2-1	175	415.377	0.3571	0.8156	2.455
2-2	175	374.979	0.3265	0.7336	20.1
2-3	18791	355.91	0.357	0.8165	2.86
2-4	18791	319.189	0.306	0.7336	2.376

## Data Availability

The data used to support the findings of this study are available from the corresponding author upon request.
